# A New York Restaurant

**Published:** 1885-12

**Authors:** 


### A NEW YORK RESTAURANT.

"I wish you would show me the most American thing in New York," said a travelling Englishman to a reporter, who, wishing to oblige, escorted him to one of the huge, always-open restaurants near a leading market at an hour when dinner was serving and the rush was heaviest

"There are a great many characteristic sights in the metropolis," said the newspaper man, "but it is doubtful whether you will find one racier of the soil than this. I hope your stomach is not sensitive, for to English nerves the truly American way of feeding the hungry is not particularly appetizing."

A clash and clatter of dishes, a roar of conversation, a fizzing accompaniment of electric lights and a bewilderment of calls that would have done credit to a Wagnerian opera assailed the ear when the seeker after information and his guide entered the main dining-hall of the big eating house, which extended from one street to the next and had seats for nearly a thousand diners Every seat was occupied and a number of customers were standing in the passage-ways waiting an opportunity to slip in wherever a vacancy occurred.

By the time the reporter had secured a couple of seats, the Englishman, very red in the face, was beginning to think of retreat. There was something in the rush and hurry, the off-hand slamming around of plates of food and the ominous shovelling it in of the average customer which made him feel decidedly queer, and the desire to escape from the heat and odor and noise grew quite strong within him. But he pluckily swallowed his heart, thought of "Rule Britannia" and determined to see the show through, even if he could not find the courage to eat very heartily. As he took the seat his eyes travelled down the long line of animals eagerly engaged upon their repasts or waiting impatiently to be served. It was a democratic line certainly. Side by side with well-dressed men were butchers from the neighboring market in their dirty jumpers, truckmen in flannel shirts, produce dealers in the well soiled garments of the farm and barnyard. Nine-tenths of the men had their hats on. Two men in seven ate with their knives and dipped the self-same knives into the common pot of butter or their teaspoons into the sugar-bowl. Nine men out of ten ate a hearty dinner inside of twenty minutes.

But the Englishman did not have much time for philosophizing, for he had not been seated half a moment before a waiter joggled his elbow, tipped over a water pitcher with apparent carelessness, but real skill, and righted it after filling a couple of goblets, whisked a bill of fare under the stranger's nose and hoarsely muttered:

"Wot'll y'ave?"

The reporter rescued the *menu* and looked the waiter in the eye.

"Make it two turtles, double porterhouse, sweet-breads, succotash, tomatoes, baked dumplings and a bottle of claret. Good shapes for a quarter," he said.

The waiter smiled, winked and was gone. Even in a democratic market restaurant the tip is an occasional visitor and a powerful ally.

"You can get a very palatable meal here, consisting of a roast or broil, with bread and butter and potatoes for fifteen cents," explained the reporter, "but I have ordered a somewhat more elaborate repast in order to show you the capabilities of this place."

Before he had finished speaking the waiter had returned with a clean table-cloth, which, by a species of legerdemain known to the craft, was quickly spread with scarcely any disturbance to the caster, water pitcher and butter dish, or to the customers eating on the other side of the table. Then came a couple of plates of turtle soup, over whose fragrant fumes the Englishman soon found his appetite, and, entering into the spirit of the place, he ate a hearty meal, unmindful of the fat market man opposite him who shovelled stewed tripe and onions, Catskill pudding, and tea into his mouth as if it were a coal hole and he was getting in his winter's fuel.

"Well, I am astonished, indeed," said the Englishman. "I do not believe I ever ate a more delicious steak in my life. It was toothsome, tender and juicy, and broiled to a turn. I imagine the Beefsteak Club could not do better. The turtle soup was as good as you will find anywhere, and those dumplings were certainly a great treat to me. We don't know much about them at home. It is certainly extraordinary that such a meal can be obtained in a place of this character. It is extraordinary to me that the class of men who would call for such cuts of beef and such soup should come here and commingle with these dirty market men and other common fellows who seem to be its mainstay."

Then it was that the reporter smiled.

"My dear sir," he exclaimed, "it's the dirty marketmen who order this class of dishes as a rule and not men in broadcloth or laundered shirts. The better dressed patrons, clerks and merchants inquire for the fifteen cent dishes, but the burly butchers and fish men and cheese men—they are the chaps that want the good living. You can't judge them by their looks. Men come in here with bloody blouses on who are worth their hundreds of thousands. They are rough fellows, perhaps grown coarse and bulky from years of contact with their quarters and halves of slaughtered animals, but they have big appetites and big pocket-books. They know what good eating is and they demand the best. They will not pay a fancy price for it, but they pay a fair price and never grumble. This huge restaurant which you see here was built upon the custom of these men. It began by furnishing six cent roasts, and gave such value for the money that custom grew apace ; new rooms were taken into the restaurant, until it had gone through the block, had extended on either side and had gone up stairs in search of additional space. The owners died millionaires, and their heirs, the present owners, would probably blush to tell where their great wealth comes from. It is no uncommon thing for this place to feed fifteen thousand people in a single day of twenty-four hours. It is always open. It knows no Sundays and no holiday.

"They must buy their meat by the carcass," said the awe-stricken Englishman.

"By the carcass! They purchase it by the car load. Vegetables they get by the wagon load. A large dairy farm is taxed to send them milk and cream and butter. Eggs they get direct from the West. A feature of this big restaurant is their fruit and berries. You will find southern strawberries and peaches here when many far more pretentious eating houses up town would hesitate to buy them. Market men like novelties and they get them. Take a look at the kitchen as we pass."

The Englishman looked into a big cavern and saw huge ranges by the score, broad stretches of "hot tables" for keeping cooked food warm, big broilers, massive ovens and great steam-heated soup kettles that a man could easily find room to drown in. A score of men and women were bustling about, cooking and carving and dish washing. The whole thing looked as if the commanding officer had gone off for a holiday and taken his system with him. But the confusion was only apparent, and from the appearing disorder was evolved the prompt and careful filling of the thousand of orders, which poured like a cataract in upon the great kitchen and its busy attendants. Truly an American product, the Englishman said, as he walked out into the street. Did he like it? On the whole, no. Leisure and quiet were an indispensable portion of his meals, and failed to find tham on the bill of fare. Such a place could not survive a month in London he said. But as a curiosity he had found it highly interesting, and he did not believe he should be hungry again that day. The food had been so good he had eaten quite beyond his usual allowance.—*New York Herald*.

